# Modification of the modified Graham patch repair for duodenal perforation using the gastrocolic ligament: Two case reports

**DOI:** 10.1016/j.ijscr.2024.110614

**Published:** 2024-11-16

**Authors:** Saamia Shaikh, Erica Kozorosky, Megha Mehta, Osama Elsawy

**Affiliations:** Department of Surgery, St. Joseph's University Medical Center, Paterson, NJ, United States of America

**Keywords:** Duodenal perforation, Graham patch, Omentum, Gastrocolic ligament, Case report

## Abstract

**Introduction:**

Gastroduodenal perforations are relatively common surgical emergencies with mortality rates as high as 40%. The Graham patch repair is one surgical approach but may need to be modified when the patient lacks viable omentum. The gastrocolic ligament can be utilized to repair these perforations for coverage.

**Presentation of cases:**

Case 1: A 77-year-old female with a complex history presented with severe abdominal pain and was found to have pneumoperitoneum on CT scan. She was found to have a first segment duodenal perforation. We employed a modified Graham patch omentopexy utilizing the gastrocolic ligament to repair the defect. She recovered well with no complications.

Case 2: A 65-year-old male with multiple myeloma presented with chemotherapy intolerance and diffuse abdominal pain. CT scan demonstrated pneumoperitoneum. Upon surgical exploration, he was noted to have a 1 cm anterior duodenal perforation. He had almost no viable omentum and therefore underwent a modified Graham patch repair using the gastrocolic ligament. He recovered well with no complications.

**Discussion:**

There have been reports of patients with gastroduodenal perforation with suboptimal omentum who underwent modified repair with the falciform ligament or a jejunal serosal patch repair. The gastrocolic ligament was found to be an effective alternative for our cases. This approach is an attractive one due to its relative ease and effectiveness.

**Conclusion:**

We described the use of the gastrocolic ligament as an alternative approach for gastroduodenal perforation in patients with suboptimal omentum. Further studies are needed to assess long term postoperative outcomes and establish best practices.

## Highlights

• Gastroduodenal perforation is a surgical emergency that can often be treated with an omental patch.
• The falciform ligament and jejunal serosal patch repair have been used as alternatives to the greater omentum.
• In select patients where the greater omentum is not viable, the gastrocolic ligament may present a viable alternative.

## 1 Introduction

Gastroduodenal perforation can be traumatic or spontaneous in nature and can be caused by several etiologies, most commonly peptic ulcer disease. According to the literature, mortality rates as high as 40 % in patients with perforated peptic ulcers have been reported [1]. The surgical treatment of these perforated ulcers was first described by Dr. Roscoe R. Graham in 1937. The original Graham patch repair described placing interrupted sutures on both sides of the defect. A portion of the greater omentum is then placed over the defect following which the previously placed sutures are tied one at a time, from superior to inferior, anchoring the vascularized omental pedicle graft in place over the perforated ulcer [2]. The modified Graham patch repair involves suturing the defect first before buttressing the repair with an omental flap. Here, we describe two patients with duodenal ulcer perforations who underwent surgical repair using a modified approach of the traditional Graham patch repair using the gastrocolic ligament. This case has been reported in line with the SCARE criteria [3].

## 2 Presentation of cases

### 2.1 1st case

The first patient is a 77-year-old female, retired teacher, with a complex medical history including Mixed Connective Tissue Disease, Systemic Lupus Erythematosus, Pulmonary Fibrosis, Sjogren's Syndrome, Scleroderma, Osteoporosis, and Hypertension, who presented with a one-day history of acute onset severe abdominal and back pain. The patient reported 10 out of 10 pain, which was primarily localized to the right upper quadrant and was not associated with nausea, vomiting, fevers, chills, or diarrhea. She denied recent trauma or prior history of peptic ulcer disease.

**Fig. 1:**
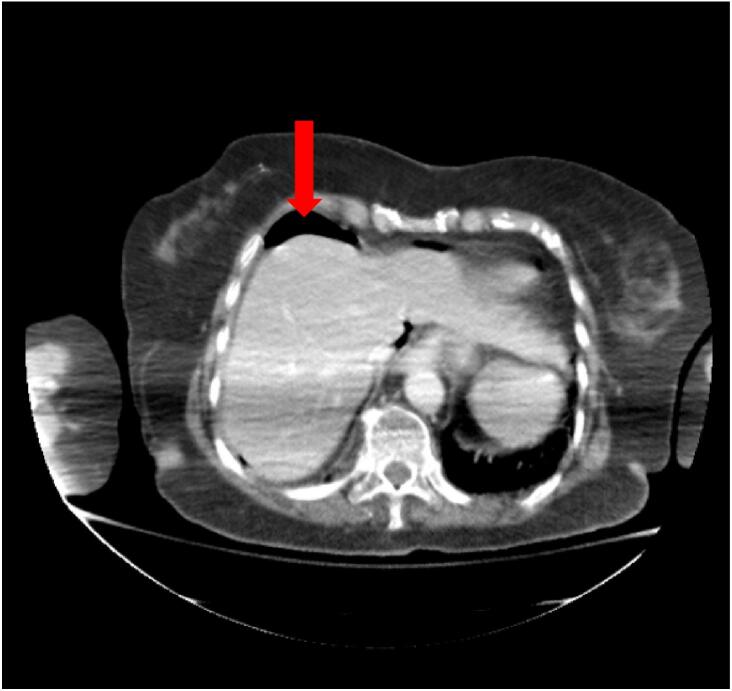
Computed tomography of the abdomen and pelvis revealing pneumoperitoneum (red arrow). (For interpretation of the references to colour in this figure legend, the reader is referred to the web version of this article.)

The patient was tachycardic and on physical examination she had peritoneal signs. She had a leukocytosis of 12,100 cells per microliter (μL). Computed Tomography (CT) of the abdomen and pelvis was obtained and revealed pneumoperitoneum in the perihepatic space. Free air was also noted below the anterior abdominal wall at the level of the umbilicus, indicative of acute bowel perforation (Fig. 1).

**Fig. 2:**
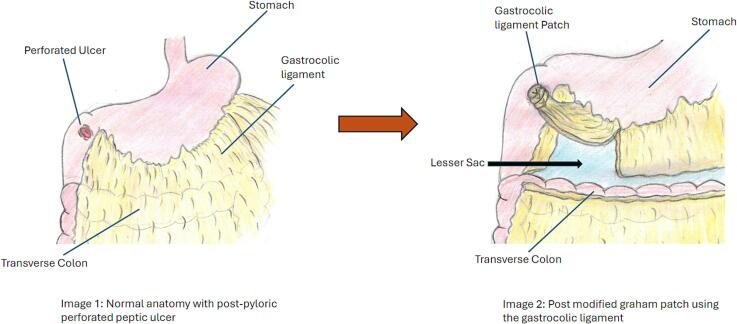
Illustration of a Modified Graham Patch Repair using the Gastrocolic Ligament.

The patient was taken to the operating room for an Exploratory Laparotomy and was found to have an approximately 7 mm anterior duodenal perforation along with free intraabdominal fluid. The lesser sac was entered by opening the gastrocolic ligament using a LigaSure device, and no free fluid or posterior gastric wall abnormalities were appreciated. A modified Graham patch was used to repair the duodenal perforation. The patient's omentum was thin and did not appear to be well-vascularized. As such, we utilized a fat pedicle from the lateral aspect of the gastrocolic ligament, which was warm, bright yellow, and obviously very well-vascularized. We decided not to use an alternative approach such as creating a flap with the falciform ligament because when the gastrocolic ligament—which was already mobilized to enter the lesser sac—was reflected cephalad, the fat pedicle sat comfortably without any undue tension over the duodenal perforation. It was sutured in place using three separate 2–0 silk sutures to create a tension-free repair (Fig. 2). A nasogastric tube (NGT) was placed by anesthesia and was palpated in the gastric lumen. An air leak test was performed by insufflating the gastric lumen while simultaneously occluding the duodenum distal to the repair, which revealed no air leak at the repair site.

**Fig. 3:**
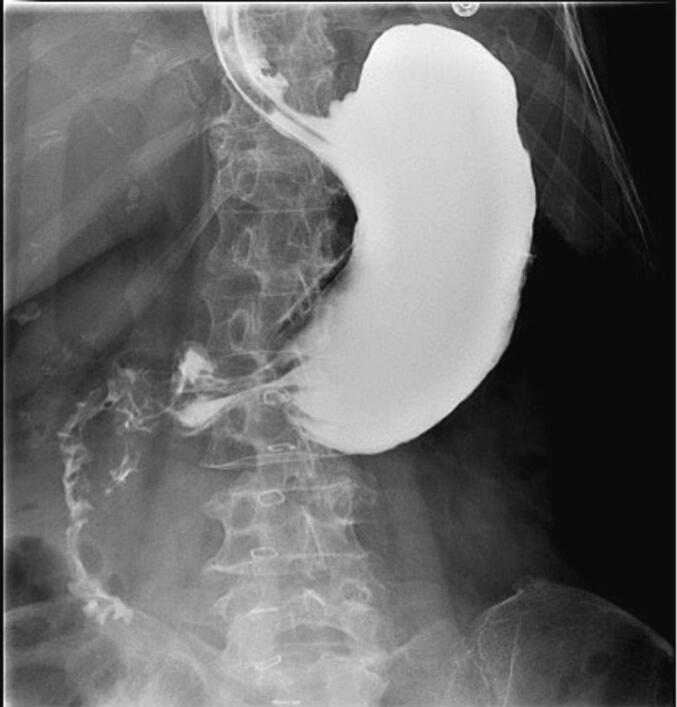
Upper GI Series without evidence of leak or obstruction after surgical repair.

The patient was kept nil per os (NPO) with a NGT in place. On postoperative day two, an upper gastrointestinal series (UGIS) was obtained and was negative for contrast leak (Fig. 3). The NGT was removed, and the patient was started on a clear liquid diet. She was discharged on postoperative day five on a mechanical soft diet. She was seen in the surgery office for follow-up three weeks postoperatively and was doing well and without complications.

### 2.2 2nd case

**Fig. 4:**
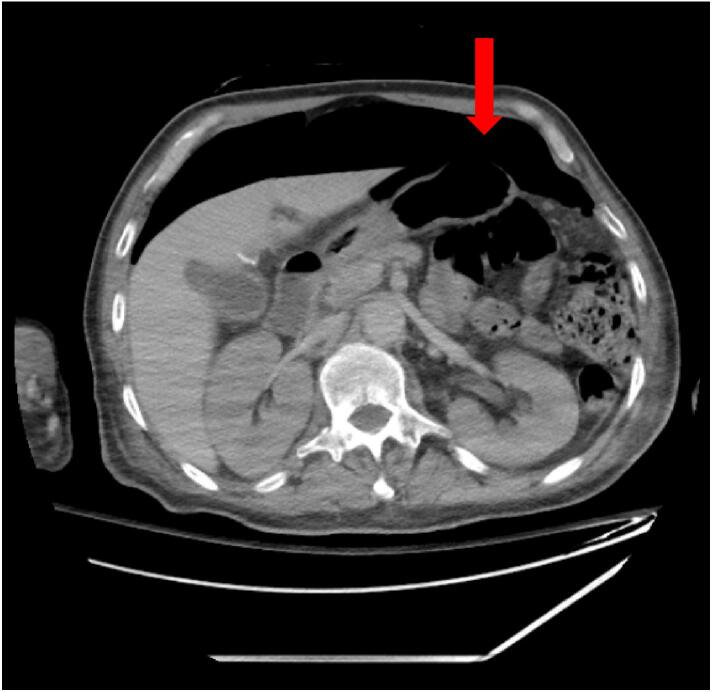
Computed tomography of the abdomen and pelvis revealing pneumoperitoneum (red arrow). (For interpretation of the references to colour in this figure legend, the reader is referred to the web version of this article.)

The second patient is a 65-year-old male with multiple myeloma status post two rounds of chemotherapy only due to intolerance, who was sent to the hospital by his oncologist for acute renal failure requiring emergent dialysis. On arrival, the patient was hypotensive and was admitted to the medical intensive care unit. On hospital day five, the patient complained of abdominal discomfort and was also found to have a rising leukocytosis of 23,100 cells/μL. On exam, patient's abdomen was soft, mildly distended, diffusely tender, but non-peritoneal. CT of the abdomen and pelvis revealed extensive pneumoperitoneum as well as free fluid in the abdomen compatible with a perforated hollow viscus (Fig. 4).

The patient was taken to the operating room emergently for an exploratory laparotomy and was found to have a 1 cm anterior duodenal ulcer perforation perforated ulcer. Bilious fluid was present in the right upper quadrant. The gastrocolic ligament was then divided using a LigaSure device and the lesser sac was entered. There were no abnormalities found on the posterior gastric wall and no fluid was present in the lesser sac. The duodenal perforation was repaired using a modified Graham patch technique. A running 3–0 silk suture was first used to repair the ulcer. The patient was noted to have almost no greater omentum to create a patch. As such, part of the mobilized gastrocolic ligament which appeared to be well- vascularized, was reflected superiorly, and was subsequently used for the patch repair. The pedicle was placed on top of the duodenal suture repair in a tension-free fashion. It was affixed in place using three separate seromuscular sutures utilizing 2–0 silk. A NGT was placed by anesthesia and was palpated in the gastric lumen. An air leak test was performed and was negative for a leak.

**Fig. 5:**
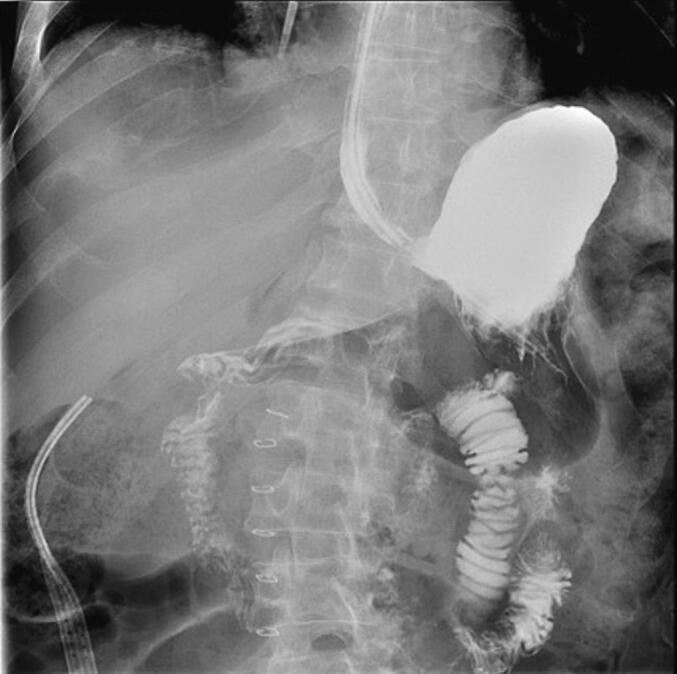
Upper GI Series without evidence of leak or obstruction after surgical repair.

Postoperatively, the patient was kept NPO with a NGT. On postoperative day two, an UGIS was obtained and was negative for contrast leak (Fig. 5). The NGT was removed, and the patient was started on a clear liquid diet. He was tolerating a regular diet by postoperative day six. The patient was again seen in the hospital approximately five months post- operatively during which time he was being treated for possible tumor lysis syndrome and pneumonia. He was doing well from an abdominal standpoint, denied any abdominal pain, and was tolerating a regular diet.

## 3 Discussion

The greater omentum is a fatty peritoneal apron that consists of four layers of visceral peritoneum (i.e., two layers folded on itself). The omentum, while once thought to be inert and an annoyance for surgeons, is now known to have a multitude of beneficial and protective properties. To name a few, it aids in controlling intra-abdominal sepsis, inflammation, tissue repair, and achieving hemostasis [4,5]. The omentum also gives rise to several peritoneal ligaments: the gastrosplenic, splenorenal, gastrophrenic and gastrocolic ligaments.

The traditional Graham patch repair involves an omentopexy utilizing a well-vascularized pedicle of the greater omentum that is placed atop of the perforated ulcer. The Graham patch repair was later modified, starting with a primary repair of the area of perforation followed by performing the omentopexy [6]. Since then, several authors have reported a similar approach using the falciform ligament instead of omentum. This was initially reported in 1978 [7]. Furthermore, while additional surgical approaches exist, such as the jejunal serosal patch repair (i.e., Thal patch), duodenostomy, pyloric exclusion, and Roux-en-Y duodenojejunostomy, the Graham patch repair remains the most widely used repair due to its relative ease and effectiveness [8]. Patients who present with acute perforation of the stomach or duodenum may be unstable and therefore performing a quick, but effective, surgical repair which does not prolong operative time is imperative.

For the repair to be successful, the omentum should be viable, well vascularized, and not strangulated. The omentopexy should be performed in a tension-free fashion and typically for small perforations less than 1 cm. These fundamentals, once followed, ensure a safe repair without risk of developing a delayed leak.

Gastroduodenal perforation can occur for a number of reasons and may be traumatic or spontaneous in nature. Most commonly, gastroduodenal perforation is due to peptic ulcer disease due to *H. pylori* or NSAID use, however, other causes of and risk factors for perforation include but are not limited to malignancy, infectious processes such as tuberculosis, autoimmune conditions such as Chron's disease or scleroderma, as well as chemotherapy and steroid use [9,10].

In some patients, including the elderly and patients with low body mass indexes or low visceral fat, this well-established repair technique may not be feasible. Similarly, patients who have previously had abdominal surgeries such as colectomies or surgery for small bowel obstruction with possible omentectomy may not have healthy omentum available. Thin, nonviable, and sometimes non-existent greater omentum may be encountered. In these cases, the falciform ligament or a Thal patch repair can provide viable alternatives to repair a perforated peptic ulcer [8].

In the case of our two patients who both had high risk factors for gastroduodenal perforation—i.e., autoimmune conditions such as Mixed Connective Tissue Disease, Systemic Lupus Erythematosus, and Scleroderma as well as steroid use in the first case, and chemotherapy and steroid use in the second case—we were able to successfully utilize the gastrocolic ligament as a viable option to perform the omentopexy with no significant post-operative complications. Although our patients did well on short term follow up, continued follow up is necessary to monitor for potential complications such as delayed leak, stricture, or abdominal abscess formation.

## 4 Conclusion

Gastroduodenal perforation is a surgical emergency that can often be treated with an omental patch. The falciform ligament and Thal patch repair have been traditionally used as alternatives to the greater omentum. In select patients where the greater omentum is not viable or cannot be juxtaposed with the area of perforation, the gastrocolic ligament may present a viable option, provided the similar fundamentals of omentopexy are followed. This paper presents only two unique operative experiences, and further studies are needed to assess the long-term outcomes and establish best practices to manage these unique patients.

## Consent

Written informed consent was obtained from the patient for publication of this case report and accompanying images. A copy of the written consent is available for review by the Editor-in-Chief of this journal on request.

## Ethical approval

This case report did not require approval by the ethics board at St. Joseph's University Medical Center.

## Guarantor

Dr. Osama Elsawy, DO, FACS.

## Research registration number

N/A.

## Funding

This research did not receive any specific grant from funding agencies in the public, commercial, or not-for-profit sectors.

## Author contribution

(1) the conception and design of the study, or acquisition of data, or analysis and interpretation of data: Osama Elsawy, Saamia Shaikh
(2) drafting the article or revising it critically for important intellectual content: Saamia Shaikh, Erica Kozorosky, Megha Mehta, Osama Elsawy
(3) final approval of the version to be submitted: Saamia Shaikh, Erica Kozorosky, Megha Mehta, Osama Elsawy

## Conflict of interest statement

The authors have no conflicts to disclose.
